# Enhanced Thermoelectric Performances of CNTs-Reinforced Cement Composites with Bi_0.5_Sb_1.5_Te_3_ for Pavement Energy Harvesting

**DOI:** 10.3390/nano12213883

**Published:** 2022-11-03

**Authors:** Hongyu Zhou, Huang Liu, Guoping Qian, Peng Xu, Huanan Yu, Jun Cai, Jianlong Zheng

**Affiliations:** National Engineering Laboratory for Highway Maintenance Technology, School of Traffic and Transportation Engineering, Changsha University of Science and Technology, Changsha 410114, China

**Keywords:** Bi_0.5_Sb_1.5_Te_3_, CNTs, cement, thermoelectric performances, energy harvesting

## Abstract

Driven by the huge thermal energy in cement concrete pavements, thermoelectric (TE) cement has attracted considerable attention. However, the current TE cement shows poor performance, which greatly limits its application. Herein, a series of Bi_0.5_Sb_1.5_Te_3_/carbon nanotubes (CNTs) co-reinforced cement composites have been prepared, and their TE properties were systematically investigated. It was shown that the addition of Bi_0.5_Sb_1.5_Te_3_ particles can effectively improve the TE properties of CNTs-reinforced cement composites by building a better conductive network, increasing energy filtering and interfaces scattering. The Bi_0.5_Sb_1.5_Te_3_/CNTs cement composites with 0.6 vol.% of Bi_0.5_Sb_1.5_Te_3_ exhibits the highest ZT value of 1.2 × 10^−2^, increased by 842 times compared to that of the CNTs-reinforced cement composites without Bi_0.5_Sb_1.5_Te_3_. The power output of this sample with the size of 2.5 × 3.5 × 12 mm^3^ reaches 0.002 μW at a temperature difference of 19.1 K. These findings shed new light on the development of high-performance TE cement, which can guide continued advances in their potential application of harvesting thermal energy from pavements.

## 1. Introduction

Cement is one of the most abundantly produced and basic structural materials, which is widely used in infrastructures, including roads, bridges and buildings [1,2,3]. Due to the low heat dissipation rate of cement, the cement-related infrastructures store large-scale thermal energy after exposure to a large quantity of sun irradiation [4,5]. This results in structural damage in the cement-related infrastructures [6], the growing intensity of urban heat island effect and increasing energy consumption of the air conditioning system in summer [7,8].

In fact, one of the best choices is using thermoelectric (TE) materials to convert large-scale thermal energy into green electricity via Seebeck effect [9,10,11,12]. It is well known that the performance of a TE material relies on the dimensionless figure of merit ZT = σα^2^T/κ, where σ, α, κ, and T are the electrical conductivity, Seebeck coefficient, thermal conductivity, and absolute temperature, respectively [13]. Good TE cement should possess high ZT value to improve the conversion capability from heat to electricity. Therefore, it is of significance to maximize the ZT value by enhancing the electrical transport properties (σ and α) and reducing thermal transport properties (κ). From the discovery of the Seebeck effect in carbon-fiber-reinforced concrete [14], numerous research studies have been carried out to explore various high ZT cement-based functional TE materials [15,16,17,18,19,20,21]. Unfortunately, the performances of most kinds of TE cement are less than 1.0 × 10^−5^ due to the poor electrical transport properties of the cement matrix.

Using carbon nanotubes (CNTs) as functional additives can significantly increase the electrical conductivity of cement because of their super σ and better conductive channel [22,23]. The σ of cement with 1 wt.% CNTs possessed a theoretical value of 3 S/m, increased by about 3000 times that of the cement without CNTs [24]. However, its α is primitive [25]. Wei et al. investigated the effect of functional additives (e.g., carbon materials [26,27] and metallic oxide [28,29]) on the TE performance of cement and found that the nano-metallic oxide increased the α. Liu et al. [30] discovered that forming a better electric conductive path was found to be very effective in tuning the σ of Bi_2_Te_3_ and carbon-fiber-reinforced cement composites. Ghosh et al. [31] reported that a graphene and ZnO nanoparticle double doping method can simultaneously increases the σ and α of cement composites. The relatively high α is due to the quantum confinement effect of the nanostructured ZnO. Besides, the κ of TE materials with multi-scale architecture is lower due to the phonon scattering at grain boundaries [32,33,34]. Therefore, the combination of multi-additives and multi-scale architecture could further improve TE performance.

Herein, we choose CNTs and multi-scale Bi_0.5_Sb_1.5_Te_3_ particles as co-reinforced phase in cement composites, taking into account that Bi_0.5_Sb_1.5_Te_3_ is the commercial *p*-type TE materials near room temperature. Remarkably, it has many attractive features, such as excellent σ, considerably large α and low κ [35]. It demonstrates that optimizing the Bi_0.5_Sb_1.5_Te_3_ contents can not only enhance the σ values of cement composites by forming a shorter conductive network, but can also adjust α and κ via the enhanced scattering with multi-scale Bi_0.5_Sb_1.5_Te_3_ particles. As a result, the largest ZT value of Bi_0.5_Sb_1.5_Te_3_/CNTs co-reinforced cement composites with 0.6% Bi_0.5_Sb_1.5_Te_3_ reaches 1.2 × 10^−2^ at 400 K, increased by 842 times compared to that of the CNTs-reinforced cement composites without Bi_0.5_Sb_1.5_Te_3_. We also present the power generation performance of the optimized Bi_0.5_Sb_1.5_Te_3_/CNTs co-reinforced cement composites. The results introduce a new promotion of the TE cement application for harvesting green electricity from pavement.

## 2. Materials and Methods

### 2.1. Raw Materials

Highly pure metals Bi (99.999%, powder), Sb (99.9%, powder) and Te (99.999%, powder) (Aladdin Biochemical Technology Co., Ltd., Shanghai, China) were used as starting materials to prepare the multi-scale Bi_0.5_Sb_1.5_Te_3_ particles. CNTs (purity > 90%, SSA > 80 m^2^/g, electrical conductivity = 69.7 S/cm) (Aladdin Biochemical Technology Co., Ltd., Shanghai, China) were used as conductive phase with lengths of 10–20 μm. Melamine sulfonated polycondensate (F10), hydroxyethyl cellulose (HEC), polyethylene glycol compound (P803), and silica fume (Shanghai Qinhe Chemical Co., Ltd., Shanghai, China) were employed as a water reducer, dispersant, defoamer, and active agent, respectively. Cement (P.O 42.5R) (Zhucheng Yangchun Cement Co., Ltd., Shangdong, China) was used as a matrix. Tap water was utilized as experimental solvent. No aggregate was taken.

### 2.2. Preparation of Bi_0.5_Sb_1.5_Te_3_/CNTs Cement Composites

The process for producing Bi_0.5_Sb_1.5_Te_3_/CNTs cement composites was as follows. 

First, multi-scale Bi_0.5_Sb_1.5_Te_3_ particles were prepared via the melt-quenching and grounding method; the detailed process was reported elsewhere [36].

Second, multi-scale Bi_0.5_Sb_1.5_Te_3_ particles and CNTs were treated by ultrasonic dispersion for 20 minimums in water to obtain Bi_0.5_Sb_1.5_Te_3_/CNTs dispersion. After that, cement, active agent, water reducer, dispersant, and defoamer were added into the dispersion, and then mixed by the mechanical stirrer of 10 minimums to form Bi_0.5_Sb_1.5_Te_3_/CNTs cement pastes. The weights of CNTs, water, active agent, water reducer, dispersant, and defoamer to cement were fixed at 1:1000, 1:2, 1:20, 1:200, 6:1000, and 1:2000. The doping content of multi-scale Bi_0.5_Sb_1.5_Te_3_ particles was controlled at the volume fractions of 0, 0.2, 0.4, 0.6, 0.8, and 1.0 vol.% to cement, respectively. Proportions for the cement pastes are listed in Table 1.

Final, the prepared cement pastes were poured into a polytetrafluoroethylene die and compacted with a vibrator to obtain dense Bi_0.5_Sb_1.5_Te_3_/CNTs co-reinforced cement composites. Then, the demolded samples were cured in air at the relative humidity of about 95% for 28 days. To eliminate the effects of pore solution on the TE properties, all Bi_0.5_Sb_1.5_Te_3_/CNTs cement composites were dried at 105 °C for 12 h after curing.

### 2.3. Characterization and Measurement

The constituent phases of the samples were determined by powder X-ray diffraction (XRD, PANalytical X’ Pert PR) using the Cu K_α_ radiation (λ = 0.15418 nm). The microstructures and chemical compositions were analyzed by using scanning electron microscopy (SEM, TESCAN Mira4) and electron probe micro-analyzer (EPMA, JEOL JXA-8230).

**Figure 1:**
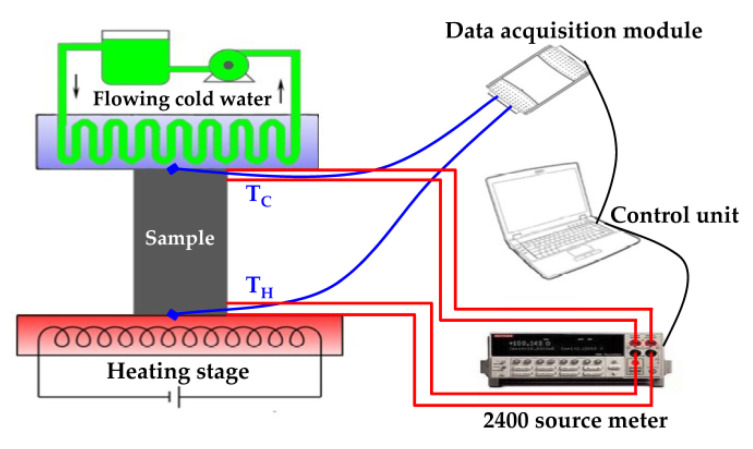
The schematic of self-made equipment for measuring properties of as-prepared samples.

The σ, α, work voltage (V), currents (I), and power output (P) of the as-prepared Bi_0.5_Sb_1.5_Te_3_/CNTs cement composites were measured by a self-made equipment, as shown in Figure 1.

The σ was given by using the equation σ = IL/VS, where L, S are the length and cross-sectional area of samples. The α was defined as α = ΔV/ΔT, where ΔV, ΔT are the open circuit voltages and temperature difference of two sides. ΔT = T_H_ − T_C_, where T_H_ and T_C_ are the hot-side and cold-side temperature. The P was calculated by using the equation P = VI. The V, ΔV and I were measured by the Source Meter instrument (SMI, Keithley 2400). The hot-side temperature (T_H_) and cold-side temperature (T_C_) were recorded by a data-acquisition module, as shown in Figure 1. During σ measurements, the T_H_ and T_C_ were fixed, and the SMI instrument was set to different workloads to measure the I and V. It was evaluated by using the two outer thermocouples for temperature (T) measurement and the inner electrodes for open circuit voltages (ΔV) measurement. During α measurements, the T_H_ and T_C_ were controlled by the heating stage and flowing cold water. When the heating stage and the flowing cold water started working, the ΔT = T_H_ − T_C_ increased. Meanwhile, the SMI instrument was connected to the samples to measure the ΔV. Thus, the σ, α, V, I, and P were obtained.

The κ was calculated using the equation κ = λρC_p_, where λ is the thermal diffusivity coefficient, C_p_ is the specific heat capacity, and ρ is the bulk density of the material. The λ was measured using a laser-flash technique (Netzsch LFA-427) with the circular block in a flowing Ar atmosphere. The C_p_ was measured using a Q20 differential scanning calorimeter. The ρ was obtained via the standard Archimedes method.

## 3. Results

### 3.1. Composition and Structure Characterization

**Figure 2:**
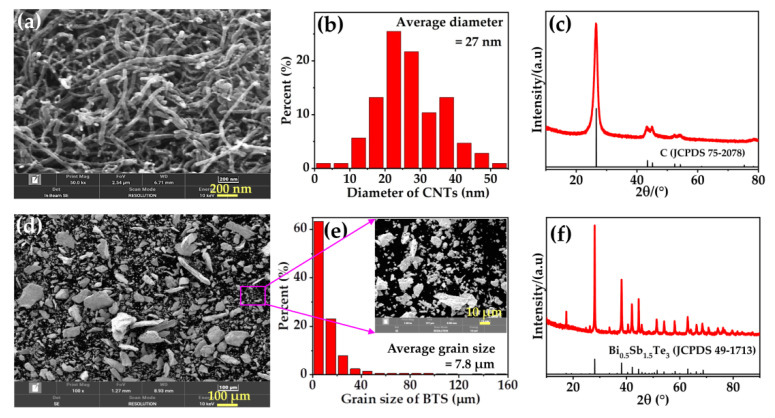
SEM images of (**a**) CNTs and (**d**) as-prepared Bi_0.5_Sb_1.5_Te_3_ powders. The corresponding size distributions, which were statistically analyzed from SEM images of (**a**,**d**), are shown along with (**b**,**e**), respectively. XRD pattern of them are also shown in (**c**) CNTs, and (**f**) as-prepared Bi_0.5_Sb_1.5_Te_3_ powders. The inset in (**e**) is the enlarged SEM image of (**d**).

Figure 2a shows the SEM image of CNTs. It is obvious that the CNTs display a tubular structure. Diameter distribution of CNTs reveals normal distribution. The average diameter of CNTs is approximately 27 nm (Figure 2b). From the XRD pattern of CNTs (Figure 2c)**,** it can be seen that all the diffraction peaks of CNTs can be indexed to the JCPDS 75-2078 file for C, which reveals that the powder is comprised of single-phase CNTs. Figure 2d and the inset of Figure 2e show the SEM image of the Bi_0.5_Sb_1.5_Te_3_ powders. It shows that the multi-scale Bi_0.5_Sb_1.5_Te_3_ particles have a wide size distribution from nm to μm. The corresponding average grain size obtained from Figure 2d is approximately 7.8 μm (Figure 2e). The XRD pattern of the Bi_0.5_Sb_1.5_Te_3_ powder shows all the diffraction peaks of the Bi_0.5_Sb_1.5_Te_3_ powders, which can be indexed to the JCPDS 49-1713 file for Bi_0.5_Sb_1.5_Te_3_ (Figure 2f). This indicates that the single phase Bi_0.5_Sb_1.5_Te_3_ has been successfully prepared.

**Figure 3:**
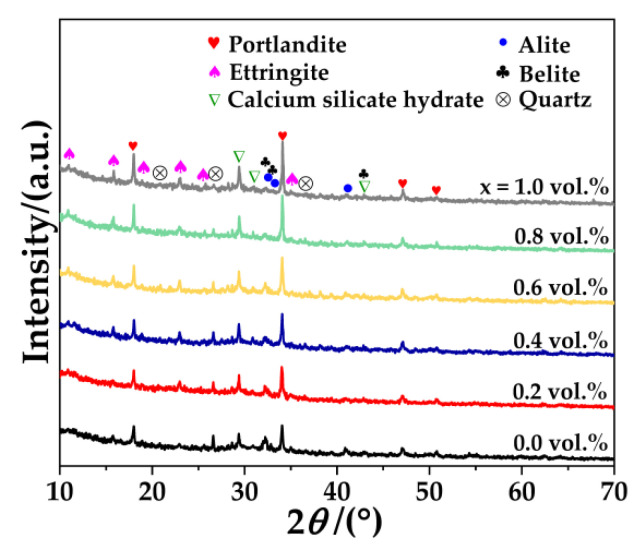
XRD patterns of Bi_0.5_Sb_1.5_Te_3_/CNTs cement composites with different Bi_0.5_Sb_1.5_Te_3_ contents.

Figure 3 displays the XRD patterns of Bi_0.5_Sb_1.5_Te_3_/CNTs cement composites with different Bi_0.5_Sb_1.5_Te_3_ contents. It can be seen that all samples consist of portlandite (Ca(OH)_2_), ettringite (AFt), calcium silicate hydrate (C-S-H gel), alite (C_3_S), belite (C_2_S) and quartz (SiO_2_). However, the diffraction peaks of Bi_0.5_Sb_1.5_Te_3_ and CNTs are not detected, because their content is lower than the detection limit of the XRD method.

It is noted that the intensities of the peaks relating to portlandite, ettringite, calcium silicate hydrate, alite, belite, and silica change. The main peak intensities of portlandite at 18.1°, 34.1° increase with the increasing contents of Bi_0.5_Sb_1.5_Te_3_. The same trends of other hydrated products (ettringite, calcium silicate hydrate) are also shown in Bi_0.5_Sb_1.5_Te_3_/CNTs cement composites, whereas the peak intensities of non-hydrated products (alite, belite and quartz) gradually decrease with the increasing contents of Bi_0.5_Sb_1.5_Te_3_, indicating that multi-scale Bi_0.5_Sb_1.5_Te_3_ particles may accelerate the hydration of Bi_0.5_Sb_1.5_Te_3_/CNTs cement composites. Similar results can be found in Ref. [37].

**Figure 4:**
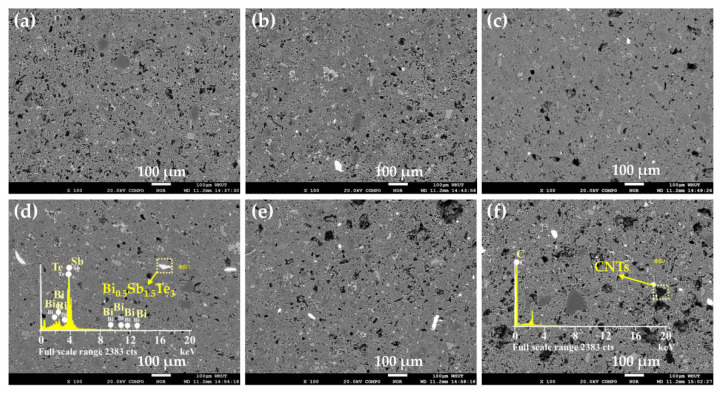
BEI photographs of Bi_0.5_Sb_1.5_Te_3_/CNTs cement composites at different Bi_0.5_Sb_1.5_Te_3_ contents: (**a**) x = 0.0 vol.%; (**b**) x = 0.2 vol.%; (**c**) x = 0.4 vol.%; (**d**) x = 0.6 vol.%; (**e**) x = 0.8 vol.%; and (**f**) x = 1.0 vol.%.

Figure 4 displays the BEI photographs of the Bi_0.5_Sb_1.5_Te_3_/CNTs cement composites. It can be seen that the brightest objects are multi-scale Bi_0.5_Sb_1.5_Te_3_ particles and the darkest objects are CNTs. More multi-scale Bi_0.5_Sb_1.5_Te_3_ particles appear when the contents of Bi_0.5_Sb_1.5_Te_3_ increase from 0.0 vol.% to 1.0 vol.%. Significantly, the CNTs are dispersed uniformly for the samples at lower Bi_0.5_Sb_1.5_Te_3_ contents, but accumulate together for the samples with the 0.8 vol.% and 1.0 vol.% Bi_0.5_Sb_1.5_Te_3_, implying that higher Bi_0.5_Sb_1.5_Te_3_ particles content (≥0.8 vol.%) may cause a serious aggregation of CNTs during the cement hydration process.

**Figure 5:**
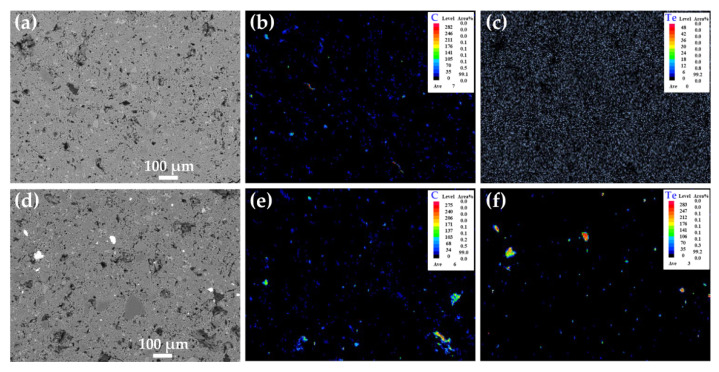
(**a**) BEI of Bi_0.5_Sb_1.5_Te_3_/CNTs cement composites at 0.0 vol.% Bi_0.5_Sb_1.5_Te_3_. The corresponding elemental maps are shown in (**b**) C and (**c**) Te; (**d**) BEI of of Bi_0.5_Sb_1.5_Te_3_/CNTs cement composites at 1.0 vol.% Bi_0.5_Sb_1.5_Te_3_. The corresponding elemental maps are shown in (**e**) C and (**f**) Te.

Figure 5 shows the backscattering electron image (BEI) and elemental maps of the Bi_0.5_Sb_1.5_Te_3_/CNTs cement composites with Bi_0.5_Sb_1.5_Te_3_ contents of 0.0 vol.% and 1.0 vol.%. The C-rich areas can be found in Figure 5b,e, suggesting that these areas contain the CNTs phase. Te-rich areas can be found in Figure 5f, indicating that these areas contain Bi_0.5_Sb_1.5_Te_3_. For the sample without Bi_0.5_Sb_1.5_Te_3_ particles, C is uniformly distributed, as shown in Figure 5b. However, when the content of Bi_0.5_Sb_1.5_Te_3_ is 1.0 vol.%, the distribution of the CNTs in Bi_0.5_Sb_1.5_Te_3_/CNTs cement composites become inhomogeneous, as shown in Figure 5e. This further reveals that the excessive Bi_0.5_Sb_1.5_Te_3_ particles can lead to the aggregation of CNTs during hydration reaction.

### 3.2. TE Properties of Bi_0.5_Sb_1.5_Te_3_/CNTs Cement Composites

**Figure 6:**
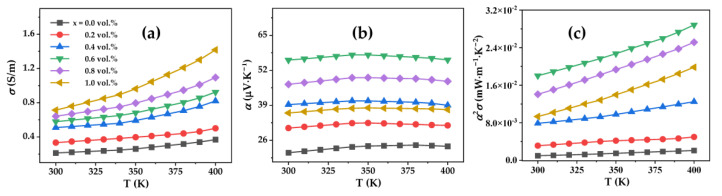
Temperature dependences of (**a**) electrical conductivity, (**b**) Seebeck coefficient, (**c**) power factor for the Bi_0.5_Sb_1.5_Te_3_/CNTs cement composites.

Figure 6a displays the electrical conductivity of Bi_0.5_Sb_1.5_Te_3_/CNTs cement composites at different Bi_0.5_Sb_1.5_Te_3_ contents. It can be seen that the σ increases with the increase in the Bi_0.5_Sb_1.5_Te_3_ content. The sample with 1.0 vol.% Bi_0.5_Sb_1.5_Te_3_ exhibits the largest σ value, which increases by 284 times compared to the sample without Bi_0.5_Sb_1.5_Te_3_.

The α of Bi_0.5_Sb_1.5_Te_3_/CNTs cement composites are shown in Figure 6b. The positive α values indicate that the majority of the carriers of the Bi_0.5_Sb_1.5_Te_3_/CNTs cement composites are holes, exhibiting p-type conduction. α first increased and then decreased in the temperature range of 300 K to 400 K as the content of Bi_0.5_Sb_1.5_Te_3_ increased. The first increase in α of the samples means that the Bi_0.5_Sb_1.5_Te_3_ multi-scale particles have a positive effect on enhancing the electrical transport properties of CNTs-reinforced cement composites. However, the α of the samples decreased with further increases in the Bi_0.5_Sb_1.5_Te_3_ contents. Combining the evolution features of microstructure from BEI results, the CNTs are uniformly dispersed at lower Bi_0.5_Sb_1.5_Te_3_ contents, but accumulate together for the samples with the Bi_0.5_Sb_1.5_Te_3_ contents of 0.8 vol.% and 1.0 vol.%. We believe that the decrease in α at the Bi_0.5_Sb_1.5_Te_3_ contents of 0.8 vol.% and 1.0 vol.% comes from a positive effect of CNTs aggregation. The sample with 0.6 vol.% Bi_0.5_Sb_1.5_Te_3_ shows the largest α of 40.66 μV/K at 350 K.

The power factor (α^2^σ) is plotted as a function of the Bi_0.5_Sb_1.5_Te_3_ content (Figure 6c), which is calculated via the measured electrical conductivity and Seebeck coefficient. A dramatic enhancement in α^2^σ is obtained in the sample with the 0.6 vol.% Bi_0.5_Sb_1.5_Te_3_ at 400 K due to the remarkable increase in α and σ. This indicates that optimizing the doping content of Bi_0.5_Sb_1.5_Te_3_ particles may effectively improve the electrical transport properties of Bi_0.5_Sb_1.5_Te_3_/CNTs cement composites.

**Figure 7:**
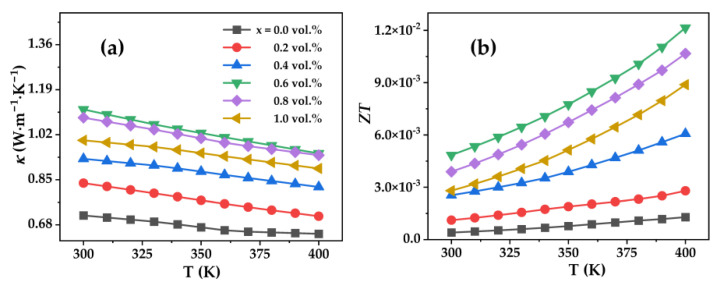
Temperature dependences of (**a**) thermal conductivity, (**b**) ZT for the Bi_0.5_Sb_1.5_Te_3_/CNTs cement composites.

The κ of Bi_0.5_Sb_1.5_Te_3_/CNTs cement composites are shown in Figure 7a. The κ of Bi_0.5_Sb_1.5_Te_3_/CNTs cement composites first increases with the increase in the content of Bi_0.5_Sb_1.5_Te_3_. However, the κ decreases with further increases in the content of Bi_0.5_Sb_1.5_Te_3_ in the range of 0.6 vol.% to 1.0 vol.%, revealing that excessive Bi_0.5_Sb_1.5_Te_3_ particles could lead to a lower κ.

As a result, the ZT values (Figure 7b) first increases and then decreases with increases in the content of Bi_0.5_Sb_1.5_Te_3_. The largest ZT reaches 1.2 × 10^−2^ at 400 K for the Bi_0.5_Sb_1.5_Te_3_/CNTs co-reinforced cement composites at 0.6 vol.% Bi_0.5_Sb_1.5_Te_3_, increasing by 842 times compared to the sample without Bi_0.5_Sb_1.5_Te_3_ particles. The dramatic enhancement in the ZT values is obtained in the sample with 0.6 vol.% Bi_0.5_Sb_1.5_Te_3_ due to the remarkable increase in σ and α. This work demonstrates that the addition of multi-scale Bi_0.5_Sb_1.5_Te_3_ particles is an effective approach to enhance the TE performance of CNTs-reinforced cement composites.

### 3.3. Evaluation of Bi_0.5_Sb_1.5_Te_3_/CNTs Cement Composites in Energy Harvesting from the Pavement

To demonstrate the potential application of the Bi_0.5_Sb_1.5_Te_3_/CNTs co-reinforced cement composites, we measure the power generation performance of Bi_0.5_Sb_1.5_Te_3_/CNTs cement composites at 0.6 vol% Bi_0.5_Sb_1.5_Te_3_ with the size of 2.5 × 3.5 × 12 mm^3^.

**Figure 8:**
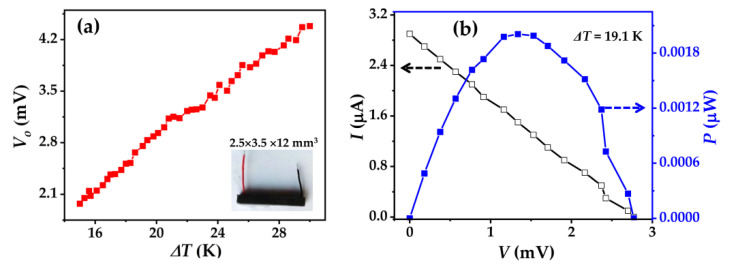
Power generation of Bi_0.5_Sb_1.5_Te_3_/CNTs cement composites at 0.6 vol.% Bi_0.5_Sb_1.5_Te_3_. (**a**) temperature difference (ΔT) dependences of open-circuit voltage (Vo) and (**b**) work voltage (V) dependences of work current (I) and power output (P) at a ΔT of 19.1 K.

Figure 8a shows temperature difference (ΔT) dependences of the generated open-circuit voltage (Vo) for Bi_0.5_Sb_1.5_Te_3_/CNTs cement composites at 0.6 vol.% Bi_0.5_Sb_1.5_Te_3_, where the cold-side temperature (T_C_) remains at 295.7 K. When the ΔT increases to 288 K and 303 K, the Vo is about 2.0 mV and 4.4 mV, respectively. Figure 8b demonstrates the work current (I) and power output (P) as a function of work voltage (V) under a ΔT of 19.1 K. The linear variation of I and the parabolic variation of P as a function of V represent a typical feature of power generators. The largest power output reaches 0.002 μW for the sample. The theoretical maximum power density P_d_ is estimated according to the equations P_d_ = P/V, where P_max_ is the maximum power output and V is the volume of the samples. From the above results, the P_d_ of Bi_0.5_Sb_1.5_Te_3_/CNTs cement composites at 0.6 vol.% Bi_0.5_Sb_1.5_Te_3_ is about 19 mW m^−3^ when the ΔT is 19.1 K.

The P_max_ value of Bi_0.5_Sb_1.5_Te_3_/CNTs co-reinforced cement composites is not higher, but the amount of cement-related infrastructure on the earth is huge. For instance, when the temperature reduction in Bi_0.5_Sb_1.5_Te_3_/CNTs co-reinforced cement concrete pavements of 1 km in length, 10 m in width and 20 cm in depth is 19.1 K, the generated electricity remains at about 38 W. With further development of TE cement, the power output of TE cement concrete pavements is likely to be enhanced, which will further promote the application of TE technology in harvesting green electricity.

## 4. Discussions

### 4.1. Conducting Behavior of Bi_0.5_Sb_1.5_Te_3_/CNTs Co-Reinforced Cement Composites

**Figure 9:**
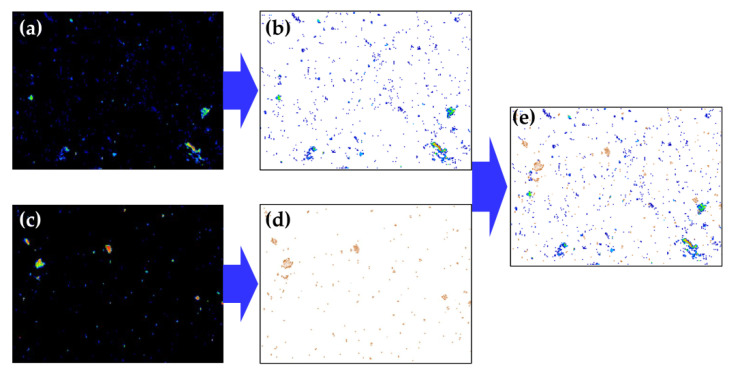
The elemental mapping of (**a**) C and (**c**) Te of Bi_0.5_Sb_1.5_Te_3_/CNTs cement composites at 1.0 vol.% Bi_0.5_Sb_1.5_Te_3_. The corresponding elemental distributions, which were extracted from (**a**,**c**), are shown in (**b**,**d**). The elemental distribution of (**d**) Te is overlayed on the elemental distribution of (**b**) C to explore the conductive network with (**e**) C+Te.

To clarify the conductive behavior of Bi_0.5_Sb_1.5_Te_3_/CNTs co-reinforced cement composites, we investigate the C and Te elemental maps of the samples with 1.0 vol.% Bi_0.5_Sb_1.5_Te_3_. The analyses are carried out by extracting C and Te distribution from their elemental maps, and then overlaying Te onto C to explore the conductive network, as shown in Figure 9. When CNTs are dispersed in cement materials, some CNTs come into contact with each other and form a local conductive network. After addition of multi-scale Bi_0.5_Sb_1.5_Te_3_ particles, a tortuous but better conductive path can be formed between Bi_0.5_Sb_1.5_Te_3_ and CNTs. Therefore, the significant enhancement in the electrical conductivity should be attributed to the occurrence of a better conductive network in Bi_0.5_Sb_1.5_Te_3_/CNTs co-reinforced cement composites.

### 4.2. Multi-Scale Bi_0.5_Sb_1.5_Te_3_ Particles and Its Effects

**Figure 10:**
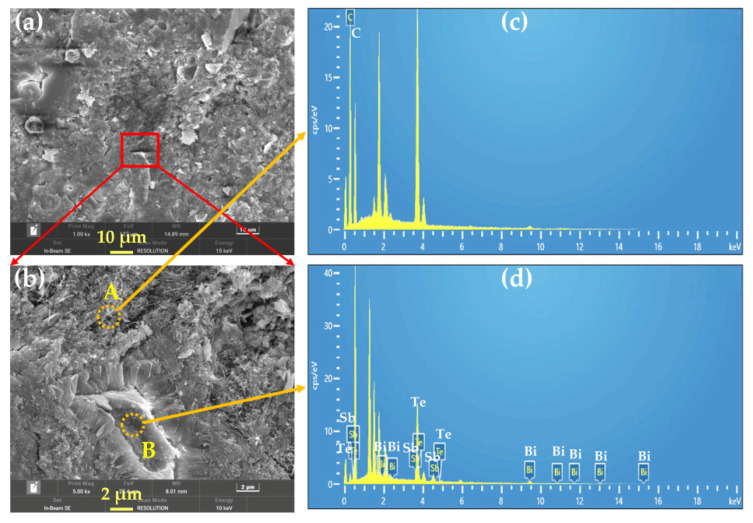
(**a**) SEM image and (**b**) enlarged SEM image of Bi_0.5_Sb_1.5_Te_3_/CNTs co-reinforced cement composites at 0.6 vol.% Bi_0.5_Sb_1.5_Te_3_. (**c**,**d**) EDS patterns of the point A and B in (**b**), respectively.

Figure 10 shows the SEM images and EDS of Bi_0.5_Sb_1.5_Te_3_/CNTs co-reinforced cement composites at 0.6 vol.% Bi_0.5_Sb_1.5_Te_3_. It can be seen that the fracture section of the samples is compact. The enlarged SEM image clearly indicates that a great variety of tube-like CNTs are distributed in the rough grain boundary of Bi_0.5_Sb_1.5_Te_3_ in cement composites, as shown in Figure 10b. The randomly bridged Bi_0.5_Sb_1.5_Te_3_ and CNTs in the co-reinforced cement composites can lead to a significant enhancement in the electrical conductivity via a better conductive network. The rough surfaces are very beneficial, decreasing the thermal conductivity by increasing the boundary scattering of phonons. In addition, the multiscale microstructures will serve as energy-dependent selective scattering centers to filter the low-energy carriers and improve the α. Hu et al. [38] observed the same phenomenon in the Bi_2_Te_3_-based TE materials. Thus, it can be concluded that the tunneling effect and phonons scattering in the co-reinforced cement composites are enhanced because of the presence of randomly arranged multi-scale Bi_0.5_Sb_1.5_Te_3_ particles, which arises from enhanced conductive network, energy-dependent selective scattering and phonon scattering in the Bi_0.5_Sb_1.5_Te_3_/CNTs-co-reinforced cement composites. 

### 4.3. Mechanism of the Enhancement in TE Performance of Bi_0.5_Sb_1.5_Te_3_/CNTs Co-Reinforced Cement Composites

**Figure 11:**
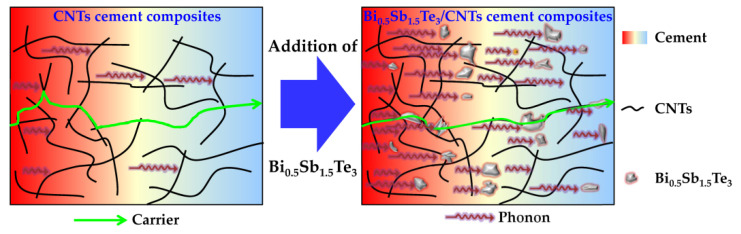
A schematic mechanism for multi-scale Bi_0.5_Sb_1.5_Te_3_ particles in enhancing the TE performance of CNTs reinforced cement composites.

To better understand the mechanism of enhanced TE performance of CNTs reinforced cement composites with multi-scale Bi_0.5_Sb_1.5_Te_3_ particles, we draw up the schematic shown in Figure 11. For cement composites, the electrical conduction involves ions, electrons, and/or holes. The main conduction of pure cement is ionic with free water solution, including Ca^2+^, Na^+^, K^+^, OH^−^, SO_4_^2−^. In this work, the cured cement composites were dried for at 105 °C for 12 h before the test. Under such circumstances, the cement paste is almost an insulating material due to no free water. When CNTs are dispersed in cement materials, some CNTs come into contact with each other to form a conductive network. The carriers (electrons and holes) will move along the conductive network or pass through the adjacent disconnected CNTs based on tunneling theory [39], which leads to an increment in σ of CNTs-reinforced cement composites. After addition of multi-scale Bi_0.5_Sb_1.5_Te_3_ particles, a tortuous but better conductive network can be formed in Bi_0.5_Sb_1.5_Te_3_ and CNTs. Hence, the electrical conductivity of Bi_0.5_Sb_1.5_Te_3_/CNTs co-reinforced cement composites is further increased.

Moreover, the multi-scale Bi_0.5_Sb_1.5_Te_3_ particles can enhance the low-energy electron filtering effect, thus improving the α of co-reinforced cement composites. However, the excessive Bi_0.5_Sb_1.5_Te_3_ particles lead to the aggregation of CNTs, thus lowering the energy-filtering effect of CNTs and α of co-reinforced cement composites. Additionally, for the multi-scale Bi_0.5_Sb_1.5_Te_3_ particles, there are a number of interfaces that can scatter the phonon, and thus lower the κ of Bi_0.5_Sb_1.5_Te_3_/CNTs co-reinforced cement composites. Consequently, the multi-scale Bi_0.5_Sb_1.5_Te_3_ particles embedded in the CNTs-reinforced cement composites increase σ and α and lower κ. The increased ZT of Bi_0.5_Sb_1.5_Te_3_/CNTs co-reinforced cement composites should be responsible for the shorter conductive network, energy filtering and interfaces scattering by the Bi_0.5_Sb_1.5_Te_3_ multi-scale particles.

## 5. Conclusions

In this paper, a series of Bi_0.5_Sb_1.5_Te_3_/CNTs cement composites were prepared via a co-reinforced method, and their TE properties were systematically investigated. It was discovered that the Bi_0.5_Sb_1.5_Te_3_/CNTs-co-reinforced cement composites with 0.6 vol.% Bi_0.5_Sb_1.5_Te_3_ exhibit the highest ZT value of 1.2 × 10^−2^. The enhanced ZT value mainly originates from the formation of shorter conductive network, increased energy filtering, and defects scattering by multi-scale Bi_0.5_Sb_1.5_Te_3_ particles. The power output of this sample with the size of 2.5 × 3.5 × 12 mm^3^ reaches 0.002 μW at a temperature difference of 19.1 K. This work introduces a successful route to develop high-performance TE cement, which can be extended to harvest thermal energy in pavements.

## Figures and Tables

**Table 1:** The proportions of Bi_0.5_Sb_1.5_Te_3_, CNTs, water, active agent, water reducer, dispersant, defoamer, and cement in the cement pastes.

	Bi_0.5_Sb_1.5_Te_3_		CNTs (g)	Water (g)	Active Agent (g)	Water Reducer (g)	Dispersant (g)	Defoamer (g)	Cement (g)
	Volume Fractions (vol.%)	Weight (g)							
B1	0.0	0.0	0.01	5	0.5	0.05	0.06	0.005	10
B2	0.2	0.043	0.01	5	0.5	0.05	0.06	0.005	10
B3	0.4	0.086	0.01	5	0.5	0.05	0.06	0.005	10
B4	0.6	0.129	0.01	5	0.5	0.05	0.06	0.005	10
B5	0.8	0.173	0.01	5	0.5	0.05	0.06	0.005	10
B6	1.0	0.216	0.01	5	0.5	0.05	0.06	0.005	10

## Data Availability

Not applicable.
